# Aberrant activation of Notch-1 signaling inhibits podocyte restoration after islet transplantation in a rat model of diabetic nephropathy

**DOI:** 10.1038/s41419-018-0985-z

**Published:** 2018-09-20

**Authors:** Yunqiang He, Mei Zhang, Ying Wu, Hemin Jiang, Hongxing Fu, Yong Cai, Ziqiang Xu, Chengyang Liu, Bicheng Chen, Tao Yang

**Affiliations:** 1 Department of Endocrinology and Metabolism, The First Affiliated Hospital of Nanjing Medical University, Nanjing, 210029 China; 2 School of Pharmacy, Wenzhou Medical University, Wenzhou, 325000 China; 3 Department of Transplantation, The First Affiliated Hospital of Wenzhou Medical University, Wenzhou, 325000 China; 4 Department of Surgery, University of Pennsylvania, School of Medicine, Philadelphia, PA 19104 USA; 5 Institute for Diabetes, Obesity and Metabolism, University of Pennsylvania, School of Medicine, Philadelphia, PA 19104 USA; 6 Key Laboratory of Surgery, The First Affiliated Hospital of Wenzhou Medical University, Wenzhou, 325000 China

## Abstract

Signaling abnormalities play important roles during podocyte injury and have been indicated as crucial events for triggering many glomerular diseases. There is emerging evidence demonstrating significant improvements in preventing renal injury and restoring podocytes after islet transplantation. However, whether signaling abnormalities affect the therapeutic efficacy of islet transplantation remain unclear. This study was established to investigate the impact of Notch-1 signaling activation on renal injury and podocyte restoration after islet transplantation. Experiments were performed in vivo and in vitro under conditions of diabetic nephropathy and high-glucose medium, respectively. Podocyte injury in vitro was induced by high-glucose concentration, and expression levels of genes associated with the Notch-1 pathway were also regulated by Jagged-1/FC and *N*-[*N*-(3,5-Difluorophenacetyl)-l-alanyl]- *S*-phenylglycine *t*-butyl ester (DAPT). Podocytes were co-cultured with islets to investigate the protective effect of islets in high-glucose conditions. Histopathological staining and transmission electron microscopy were performed to assess pathological changes in podocytes in glomeruli. The results from this study showed that Notch-1 signaling in podocytes was significantly decreased by functional islet cells in vivo and in vitro. Compared with the co-cultured group and transplanted group, highly activated Notch-1 signaling significantly moderated the effect of islets in affecting podocyte restoration and renal injury. Renal damage and podocyte injury were alleviated after DAPT treatment. Furthermore, the balance between apoptosis and autophagy was diverse under different treatments. All the data in this study showed that highly activated Notch-1 signaling could affect the therapeutic efficacy of islet transplantation on renal injury and podocyte restoration in high-glucose conditions. The balance between apoptosis and autophagy was also closely associated with the degree of podocyte restoration. This finding may suggest that the in vivo microenvironment plays a critical role in podocyte restoration after islet transplantation, which provides a promising and individual assessment and targeting treatment for different diabetic nephropathy patients after islet transplantation into the future.

## Introduction

Diabetic nephropathy (DN) is a chronic microvascular complication in diabetic patients and has become the leading cause of end-stage renal disease worldwide^1,2^. Progressive proteinuria is one of the most notable hallmarks of DN, followed by renal injury and glomerular filtration damage^3^. Podocytes are considered the major components of the filtration barrier, and persistent proteinuria is closely associated with their injury and deletion in various glomerular diseases^4^. Recent studies indicate that the restoration of podocyte structure and function maybe important for the treatment of DN^5–7^, but the outcomes remain unchanged when using the current conventional therapies. Thus, early protection and targeted therapies relating to podocyte injury are crucial in the treatment of DN patients.

Pancreatic islet transplantation has emerged as a promising alternative therapy to conventional treatments for type 1 diabetic patients and type 2 diabetic patients with complications^8,9^. Studies have reported that DN can be prevented in early diabetic patients by a functional islet graft, and pathological changes are reversed over time after transplantation^10^. Our previous studies displayed that such a treatment demonstrates a significant effect on renal damage and injury of podocytes^11,12^. However, we also found in several islet-transplanted DN rats, there was no significant improvement in renal microstructure and no improved restoration of podocytes despite blood glucose levels that were maintained in the normal range. Meanwhile, aberrant activation of Notch-1 signaling was detected in the kidneys of these rats. Emerging evidence also demonstrates abnormalities in activation of signaling pathways play a critical role during the processes causing podocyte lesions in high blood glucose conditions^13^. However, it is unknown whether aberrant activation of signaling affects podocyte restoration after islet transplantation.

The Notch-1 pathway is an evolutionarily conserved signaling pathway that controls cellular differentiation during the development of a multicellular organism^14^. Its aberrant activation in mature kidneys is considered an important mechanism in the progression of proteinuria caused by glomerular injury and podocyte lesions^15,16^. Elevated amounts of the Notch intracellular domain (NICD) in a DN environment can translocate into the nucleus and upregulate the expressions of the hairy and enhancer of split (Hes) transcription factors and vascular endothelial growth factor (VEGF), which can lead to podocyte apoptosis in the glomerulus^17^. Therefore, downregulating activated Notch-1 signaling is crucial for podocyte restoration during DN treatments^18^. Autophagy and apoptosis are two different kinds of approaches to clear damaged or aging cells and organelles to maintain intracellular integrity and homeostasis. The balance between them is also crucial during podocyte injury and restoration. It is also worth studying whether aberrant activation of Notch-1 signaling and its downstream gene-expression cascade both affect podocyte restoration by regulating the levels of apoptosis and autophagy.

This study was designed to investigate whether aberrant activation of Notch-1 signaling in the kidney affected the ameliorative efficacy of islet transplantation on renal injury and podocyte restoration. The protective effects of functional islets on podocyte injury were also evaluated in vivo and in vitro in conditions associated with high-glucose (HG) concentrations. Furthermore, whether the levels of apoptosis and autophagy in the glomerulus could be regulated by activated Notch-1 signaling was measured in this study, and the significance of microenvironment in vivo and balance between apoptosis and autophagy were also discussed in conditions associated with DN, which may provide a basis for the development of targeted treatments and assessment of individuals in clinical islet transplantations in various diabetic patients.

## Results

### Notch-1 signaling was activated only when podocyte injury reached a certain level under HG condition

**Fig. 1:**
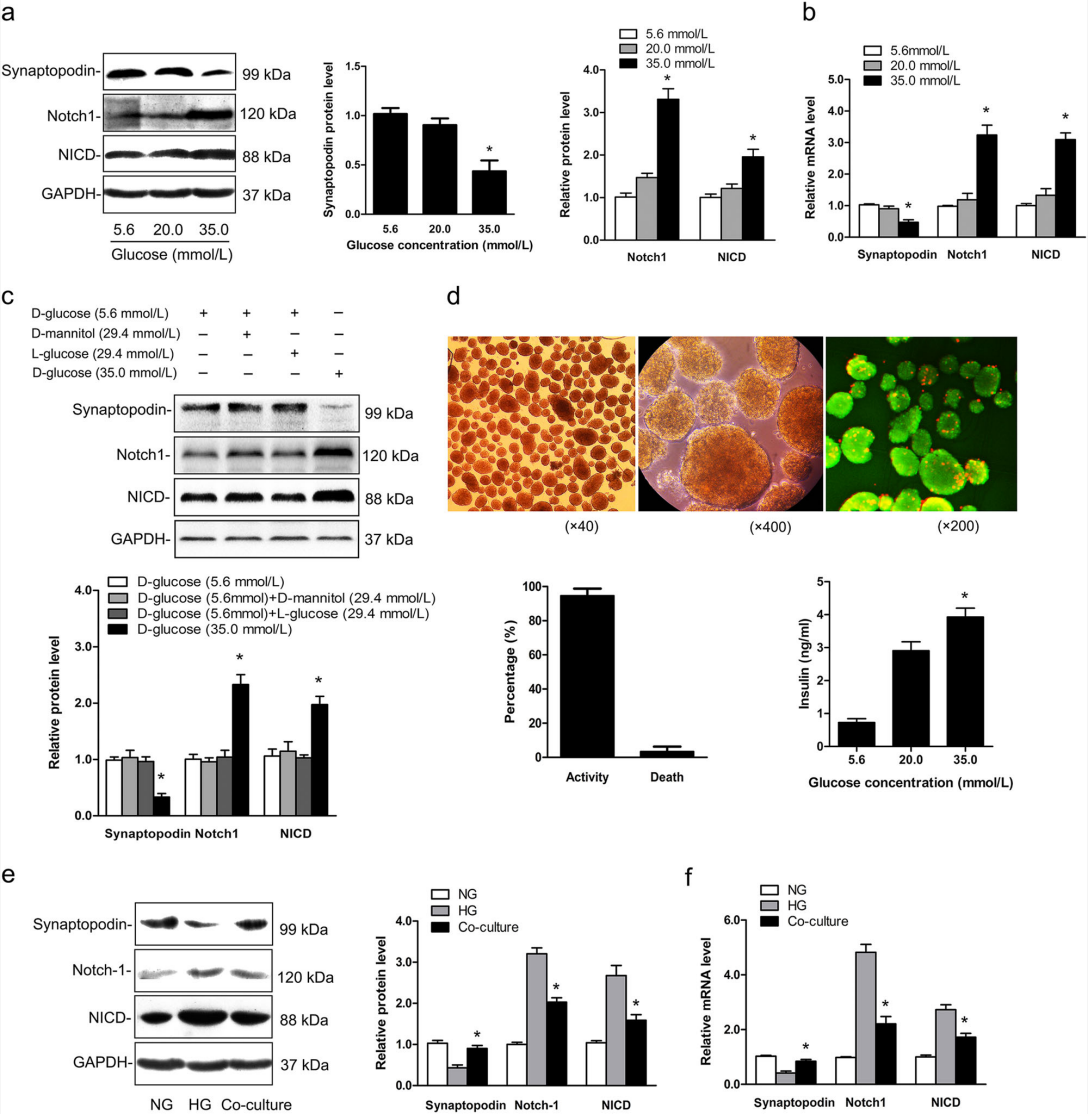
Notch-1 signaling in podocyte was downregulated when co-cultured with islets under the HG condition. **a** Notch-1 signaling expression increased significantly at 35.0 mmol/L glucose concentration, whereas it was not evident at 20.0 mmol/L (^*^P < 0.05). **b** Relative mRNA expression levels of synaptopodin, Notch-1 and NICD in podocytes under different glucose concentrations. **c** The osmotic control (d-mannitol and l-glucose) to the podocytes treated with HG in vitro (^*^P < 0.05). **d** Isolated pancreatic islets under optical microscope (×40 and ×400) and activity evaluation of islets (FDA (green) and PI (red) staining, ×200). The levels of insulin secretion of islets were measured in different glucose concentrations. **e** Podocytes were co-cultured with isolated islets, and expression levels of synaptopodin, Notch-1 and NICD were detected for each group (^*^P < 0.05 versus the HG group). **f** Relative mRNA expression levels of these proteins in different groups (^*^P < 0.05 versus the HG group). All the data are presented as the mean ± SD and Student’s *t* test was used for the comparisons between two groups

To explore whether Notch-1 signaling activation was related to the degree of podocyte injury, immortalized mouse podocytes were cultured in different glucose concentrations (5.6, 20.0, and 35.0 mmol/L). The expression levels of synaptopodin (podocytes-specific marker) and proteins related to the Notch-1 pathway were measured by western blotting and real-time PCR analysis; the results (Fig. 1a, b) showed that activated Notch-1 (NICD) signaling was accompanied with a decreased presence of podocytes at 35.0 mmol/L glucose concentration compared with the NG-treated group (5.6 mmol/L, *P* < 0.05). However, podocyte injury was not evident in the group treated with 20.0 mmol/L glucose. Also, the results of osmotic control (both d-mannitol and l-glucose) to the experiments with HG treatment showed that osmotic pressure had no significant effect on podocyte expression and Notch-1 signaling pathway activation (Fig. 1c). Thus, Notch-1 signaling activation may be closely associated with the degree of podocyte injury in HG conditions, and it was only initiated as podocytes were exposed a sufficient stimulus.

### Downregulated Notch-1 signaling in podocytes was found when they were co-cultured with islets in HG conditions

Highly purified pancreatic islets were isolated from donor rats (Fig. 1d). The activity was measured by an aliquot of islets using fluorescein diacetate-propidium iodide staining and the results showed that islets’ activity was at a high level (>95%). The insulin secretion capacity of islets was also diverse in different glucose concentrations (Fig. 1d), and it was highest at the 35.0 mmol/L glucose concentration. Then, in order to evaluate the protective effects of isolated islets on podocytes an in vitro cell co-culture experiment was performed at a HG concentration (35.0 mmol/L). As shown in Fig. 1e, f, compared with the HG group Notch-1 signaling was significantly decreased in the co-cultured group, and synaptopodin expression was higher when islets were present (*P* < 0.05). This finding suggested that functional islets may protect podocytes from injury in HG conditions, and may inhibit aberrant activation of the Notch-1 signaling pathway.

### Inhibition and activation of the Notch-1 pathway in podocytes in HG conditions

**Fig. 2:**
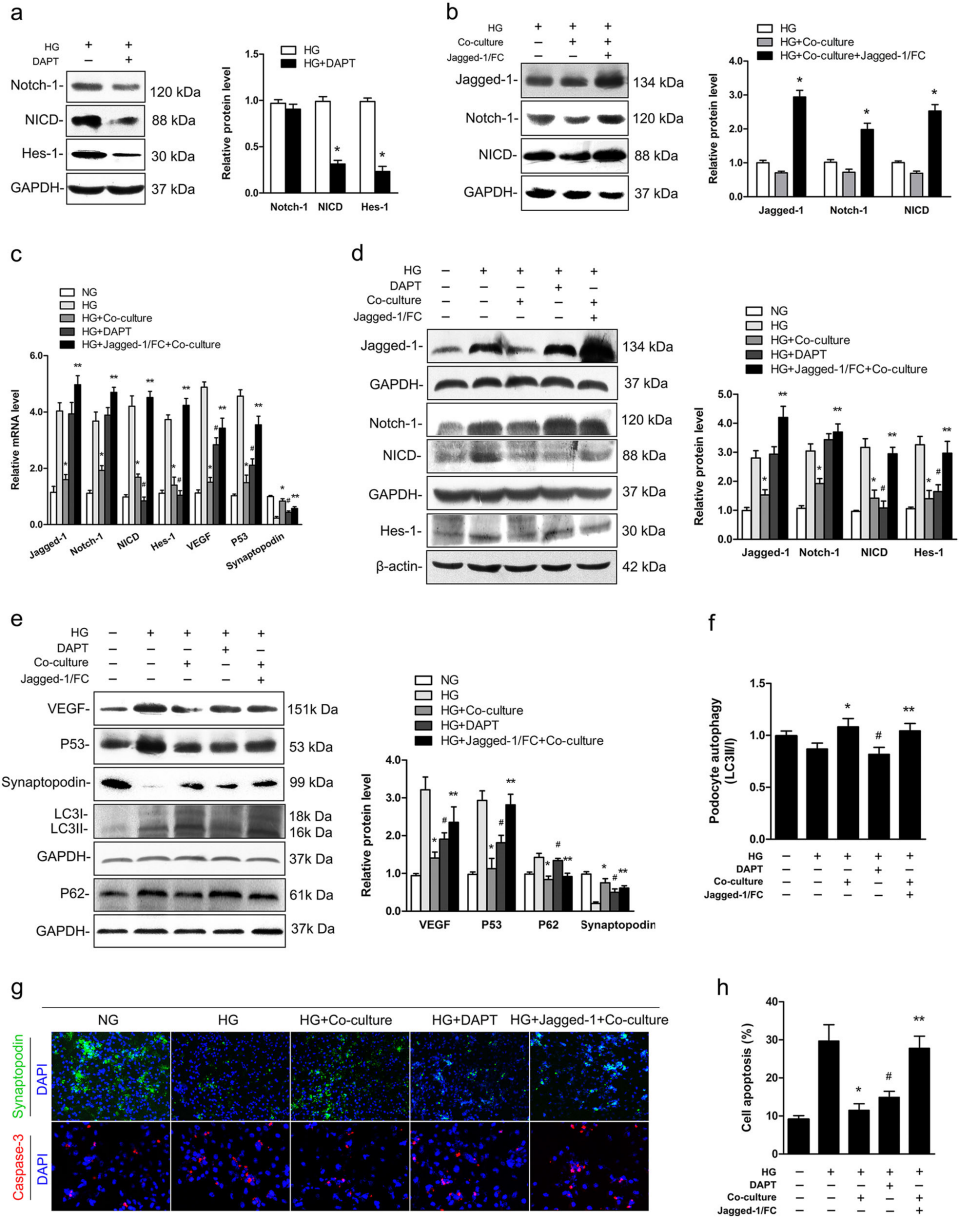
Highly activated Notch-1 signaling weakened the protective efficacy of co-cultured islets under HG condition in vitro. **a** Notch-1 signaling pathway in podocytes was downregulated by DAPT under the HG condition (^*^P < 0.05 versus the HG group). **b** Jagged-1/FC chimera could maintain Notch-1 signaling expression at a high level in podocytes when co-cultured with islets under the HG condition (^*^P < 0.05 versus the co-cultured group). **c**, **d** Quantifications of proteins expression in the Notch-1 pathway through real-time PCR and western blotting analysis (^*^P < 0.05 versus the HG group, ^#^*P* < 0.05 versus the HG group, ^**^P < 0.05 versus the co-cultured group. Owing to DAPT being a γ-secretase inhibitor of Notch-1 translocated into intracellular domain, the expression levels of Jagged-1 and Notch-1 were not analyzed in the DAPT group). **e-f** The expression levels of target genes of Notch-1 signaling pathway and autophagy-related proteins were measured for each group. **g** Representative confocal microscopic images and quantifications showing the expressions of synaptopodin (green, ×100) and caspase-3 (red, ×400) in podocytes in different groups. **h** The levels of podocyte apoptosis were analyzed for each group (^*^P < 0.05 versus the HG group, ^#^*P* < 0.05 versus the HG group, ^**^P < 0.05 versus the co-cultured group)

A γ-secretase inhibitor (*N*-[*N*-(3,5-Difluorophenacetyl)-l-alanyl]- *S*-phenylglycine *t*-butyl ester, DAPT) was used in this study to evaluate the links between podocyte injury and Notch-1 signaling in HG conditions. As shown in Fig. 2a, the results revealed that DAPT could successfully inhibit the activation of Notch-1 pathway (NICD) in podocytes in HG conditions. The previous results had demonstrated that Notch-1 signaling in podocytes was markedly reduced when they were co-cultured with islets in HG conditions. Thus, a sufficient recombinant Jagged-1/FC chimera was used as an activator of Notch-1 signaling pathway to evaluate whether sustained high levels of Notch-1 signaling activation affected protective effects of co-cultured islets on podocytes. The results (Fig. 2b) showed expression of proteins in the Notch-1 signaling pathway remained high using the combined treatments of a Jagged-1/FC chimera and co-culture of islets.

### Aberrant activation of Notch-1 signaling affected the protective efficacy of co-cultured islets on podocytes

Real-time PCR and western blotting analysis were used to measure the links between Notch-1 pathway activation and podocyte injury under different treatments (Fig. 2c, d). The results showed that Notch-1 signaling expression in podocytes was significantly activated in HG conditions. Compared with the HG group, the levels of downstream proteins in the Notch-1 pathway (NICD and Hes-1) were decreased in the co-cultured group and DAPT-treated group. However, these proteins were still at high levels in the co-culture + Jagged-1/FC group when compared with the group where just were co-cultured with islet cells. Furthermore, expression levels of p53, VEGF and autophagy-related proteins (LC3 II/LCI and P62) were also detected in this study, and results revealed that expression levels of p53 and VEGF were decreased both in the islets co-cultured group and DAPT group (Fig. 2e). In the co-culture + Jagged-1/FC group, however, expression of these proteins remained at high levels compared with the group where just islets were co-cultured (*P* *<* 0.05). As shown in Fig. 2e, f, the level of autophagy (LC3 II/LC3 I) was significantly improved in the co-culture groups (both co-culture group and co-culture + Jagged-1/FC group), but it remained low in the DAPT-treated group. Accordingly, the expression of P62 was decreased in the co-culture groups. In Fig. 2g, the results from immunofluorescence staining demonstrated that podocyte expression was at a high level when islets were co-cultured in HG conditions. However, the aberrant activation of Notch-1 signaling affected the protective efficacy of islets co-culture when treated by using the Jagged-1/FC chimera. The overall level of apoptosis was also markedly decreased in the co-culture group and DAPT-treated group compared with the HG group and Jagged-1/FC-treated group (Fig. 2g, h). This finding demonstrated that highly activated Notch-1 pathway could increase podocyte apoptosis and affect protective efficacy of islets on podocytes, but it did not affect autophagy.

### Activated Notch-1 pathway affects the improvement of renal function and microstructure after islet transplantation in DN rats

**Fig. 3:**
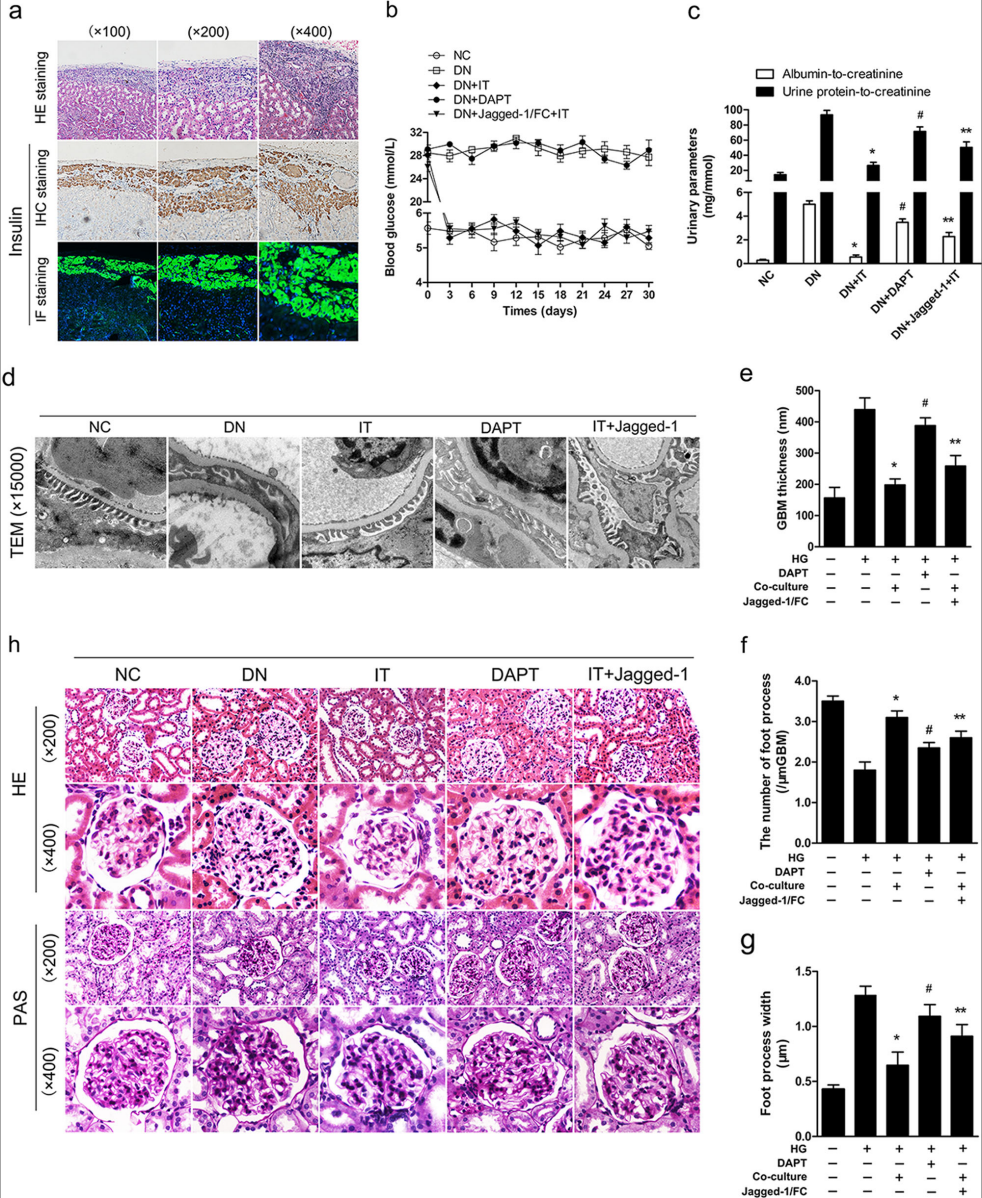
Activated Notch-1 signaling affected the improvement of renal function and microstructure after islet transplantation **a** Photomicrographs (HE staining, immunohistochemical, and immunofluorescence staining) showing transplanted islet grafts under the kidney capsule were at a high activity and still capable of normal insulin secretion. **b** Nonfasting blood glucose levels were detected for each group. **c** Urinary parameters (albumin-to-creatine and urine protein-to-creatinine) was measured for each group (^*^P < 0.05 versus the DN group, ^#^*P* < 0.05 versus the DN group, ^**^P < 0.05 versus the IT group). **d** Photomicrographs of transmission electron microscopy (TEM) showing the pathological changes of microstructure in the kidney for each group ( ×15,000). **e–g** Quantifications of mean thickness of glomerular basement membrane (GBM), number of foot process and mean foot width were measured in different groups (^*^P < 0.05 versus the DN group, ^#^*P* < 0.05 versus the DN group, ^**^P < 0.05 versus the IT group). **h** Pathological staining (HE staining and PAS staining) showing typical changes in the glomerular structure for each group

Previous studies had revealed the ameliorative effects of islet transplantation on DN rats. Pancreatic islets were isolated and transplanted into the space under the kidney capsule of DN rats (Fig. 3a). In this study, the levels of blood glucose and urinary parameters (ratios of albumin-to-creatinine and urine protein-to-creatinine) of rats were also measured in different treatments. As shown in Fig. 3b, c blood glucose levels were significantly decreased and were maintained at a normal level (5.42 ± 0.56 mmol/L) after islet transplantation in the IT (islet transplantation) group (5.40 ± 0.47 mmol/L) and in the Jagged-1/FC + IT group (5.45 ± 0.38 mmol/L), and renal injury were also markedly alleviated after islet transplantation according to urinary parameters. However, in our study, we found sustained high levels of Notch-1 signaling in the Jagged-1/FC + IT group that affected the therapeutic efficacy of islet transplantation on ameliorating renal injury in DN rats (*P* < 0.05, Fig. 3c). In addition, there was a slight decrease in the proteinuria level in DN rats with DAPT administration compared with untreated DN rats (*P* < 0.05). As shown in Fig. 3d–g, when compared with the rats in the IT group, the results from transmission electron microscopy demonstrated that renal microstructure injuries, such as thickening of glomerular basement membrane, podocyte deletion, and foot process fusion, were more evident in the Jagged-1/FC-treated rats, although they were treated with islet transplantation. Histopathological staining (HE and Periodic acid–Schiff staining) was also performed to examine glomerular pathological changes in each group. As shown in Fig. 3h, glomerular lesions were markedly alleviated after transplantation (both in IT group and Jagged-1 + IT group), but the improvement in the DAPT-treated group was not evident in this study. This finding may suggest that aberrant Notch-1 signaling activation and other damaging factors associated with diabetes (such as hyperglycemia and hemodynamic changes) in DN conditions may play different roles in affecting the process of glomerular injuries in vivo.

### Highly activated status of Notch-1 signaling inhibited podocyte restoration after islet transplantation

**Fig. 4:**
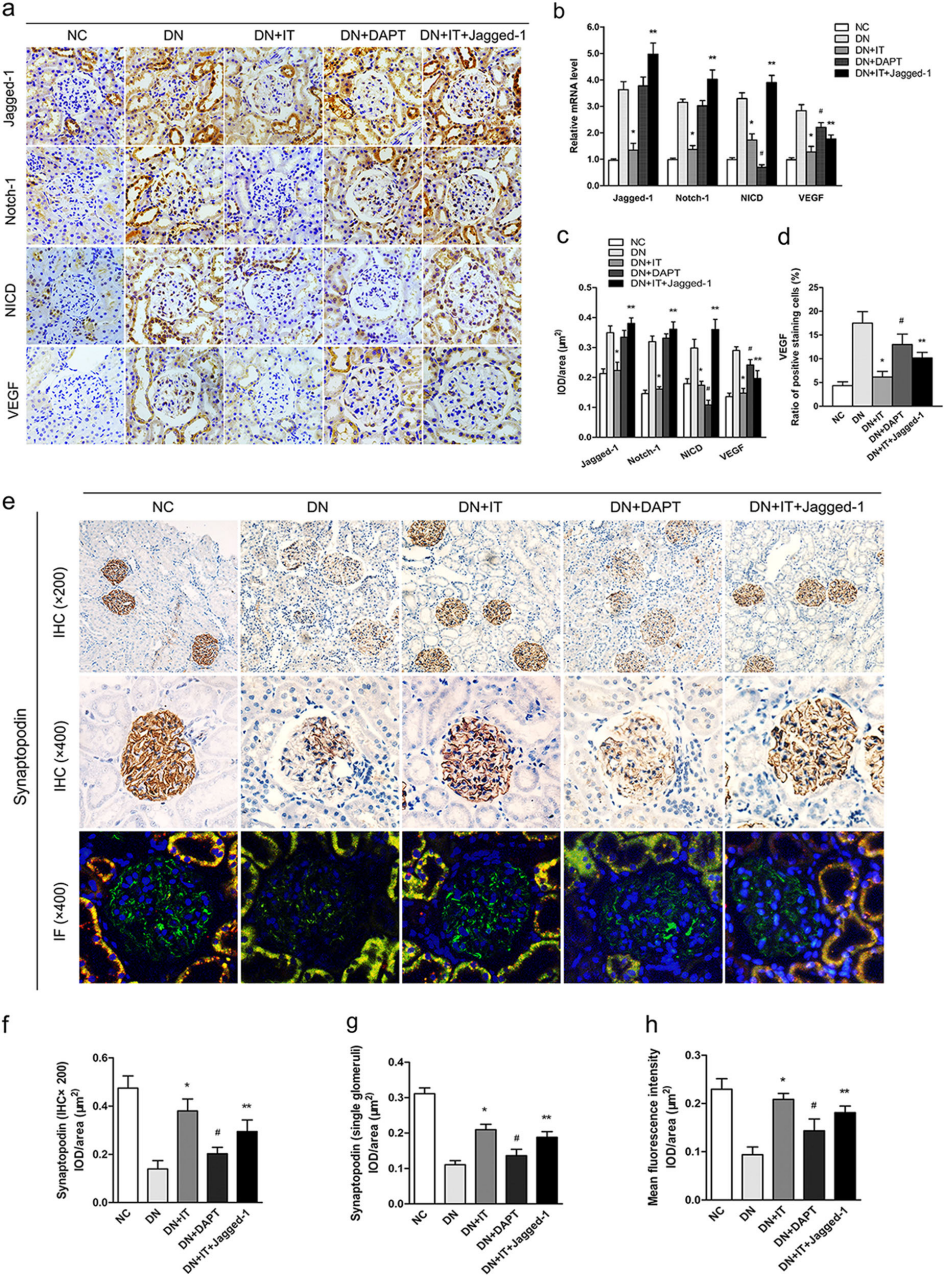
Highly activated status of Notch-1 signaling pathway inhibits podocyte restoration after islet transplantation **a–b** Photomicrographs (immunohistochemical staining) and quantifications (real-time PCR) showing the expressions and typical distribution of proteins of Notch-1 signaling pathway in different groups of kidney (^*^P < 0.05 versus the DN group, ^#^*P* < 0.05 versus the DN group, ^**^P < 0.05 versus the IT group). **c** Quantifications of these proteins expression in the kidney were measured by using the mean integrated optical density (IOD)/area with Image-Pro Plus 6.0 image analysis software. **d** The ratio of positive staining (VEGF) cells in the glomerular was detected for each group. **e** Immunohistochemical staining of podocytes and immunofluorescence staining (green: synaptopodin (podocytes); orange: E-cadherin (renal tubular epithelial cells) were performed to measure the degree of podocyte restoration in different groups. **f–h** Representative quantification of podocyte expression in single glomeruli was measured with IOD/area in immunohistochemical staining and mean fluorescence intensity for each group

Immunohistochemical staining was performed to observe the expression and distribution of proteins in the Notch-1 pathway in the kidney. As shown in Fig. 4a–c, Notch-1 signaling activation was found to be activated both in renal tubular epithelial cells and glomerular cells in DN rats. The expression of NICD was markedly reduced in the DAPT-treated group and the IT group under DN conditions. This finding also revealed that Jagged-1/FC chimera treatment could maintain Notch-1 activation at a high level in DN rats even treated by islet transplantation. The protein VEGF has been reported to be closely related to podocyte deletion, and could be upregulated by Notch-1 signaling activation in many glomerular diseases. As shown in Fig. 4d, the ratio of cells that stained positive for VEGF in the glomerulus was slightly decreased in the DAPT-treated group compared with the DN group, but it was higher than what was observed in the Jagged-1/FC group. In addition, when compared with the IT group, the ratio of VEGF-positive cells was significantly increased in the Jagged-1/FC group (*P* < 0.05). This finding may suggest that sustained increased activation of Notch-1 signaling in vivo may affect the ameliorative effects of islet transplantation. Immunohistochemical and immunofluorescence staining of podocytes in the kidney were both performed to evaluate the restoration of podocytes after different treatments in this study (Fig. 4e). The results demonstrated that podocyte levels were significantly upregulated in the IT group compared with untreated DN rats (*P* < 0.05). However, the expression level in the IT + Jagged-1/FC group was lower than the rats treated with islet transplantation alone (IT group, *P* < 0.05), which suggested that sustained high activation of the Notch-1 pathway may inhibit the restoration of podocyte structure and expression in DN rats after islet transplantation. We also found podocyte expression was slightly increased in the group treated with DAPT when compared with the DN group (Fig. f-h, *P* < 0.05).

### Activated Notch-1 pathway weakened the effect of islet transplantation on improving podocyte apoptosis but did not affect autophagy

**Fig. 5:**
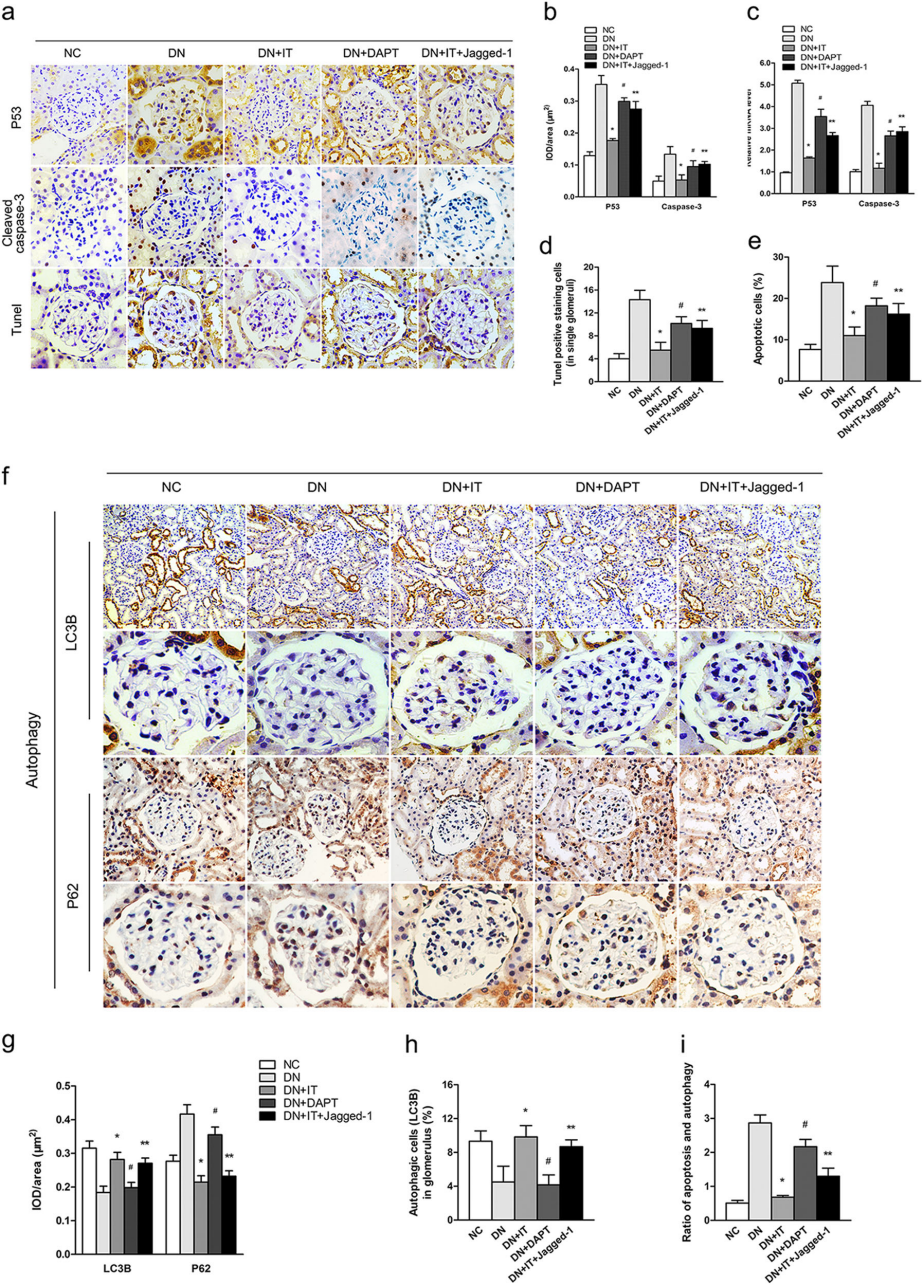
The expression level of Notch-1 signaling affected the balance between apoptosis and autophagy. **a-c** Photomicrographs and quantifications showing the expressions of P53 and caspase-3 in different groups (^*^P < 0.05 versus the DN group, ^#^*P* < 0.05 versus the DN group, ^**^P < 0.05 versus the IT group). **d**, **e** TUNEL staining was performed to analyze the level of podocyte apoptosis in single glomeruli for each group. **f–h** Representative photomicrographs and quantifications showing the expressions of autophagy-related proteins (LC3B and P62) in glomerulus in the kidney, and the level of podocyte autophagy was measured by LC3B in single glomeruli in different groups (^*^P < 0.05 versus the DN group, ^#^*P* > 0.05 versus the DN group, ^**^P > 0.05 versus the IT group). **i** The ratio between apoptosis and autophagy in single glomeruli was measured for each group

Apoptosis and autophagy play important roles in the processes associated with podocytes’ injury and restoration. For investigating whether highly activated Notch-1 signaling in the kidney affected the therapeutic efficacy of islet transplantation on podocyte restoration by regulating apoptosis and autophagy expression in vivo, terminal deoxynucleotidyl transferase dUTP nick end labeling, immunohistochemical staining and real-time PCR of apoptosis proteins (P53 and cleaved caspase-3) and autophagy-related proteins (LC3B and p62) were both performed. As shown in Fig. 5a–e, the results demonstrated that apoptosis levels in renal tubular epithelial cells and glomerular cells were significantly decreased in the IT group when compared with untreated DN rats. In addition, the expression levels of apoptosis-related proteins in the IT + Jagged-1 group were markedly higher than those observed in the IT group. Compared with the DN group, there was also a slight decrease in apoptosis-associated proteins in the DAPT-treated group (*P* < 0.05). However, the staining for autophagy-related proteins (LC3B and p62) presented a different and interesting result in this study (Fig. 5f–h). We found that the overall level of autophagy was decreased in DN conditions (DN group), but significantly increased after islet transplantation (both in IT group and IT + Jagged-1 group). In addition, there was no evident restoration of autophagy in podocytes in the DAPT-treated group. This may suggest that, in addition to activation of the Notch-1 pathway, other factors associating with diabetes in the DN condition affected autophagy in podocytes. Furthermore, the ratio of apoptosis and autophagy was also measured in our study (Fig. 5i), and we found that it was decreased significantly in DN rats after islet transplantation, which was almost maintained at normal levels. Highly activated Notch-1 signaling weakened the improvement of podocyte injury after treatment. This ratio may be closely associated with the degree of podocyte injury and restoration in many glomerular diseases.

## Discussion

This study was designed to investigate whether aberrant activation of Notch-1 signaling in the kidney affected the ameliorative effect of islet transplantation on renal injury and podocyte restoration. The results demonstrated that activation of Notch-1 signaling was closely related to the degree of podocyte injury in HG conditions. Its significant activation was only initiated as podocytes were exposed to a strong enough stimulus, and the injury of podocytes reached a certain level in HG environments. Activation of the Notch-1 pathway was markedly increased at a glucose concentration of 35.0 mmol/L, whereas this increased activation was not evident at a glucose concentration of 20.0 mmol/L. Notch-1 activation was both significantly decreased when treated with a transplant of functional islet cells in vivo (islet transplantation) and in vitro (co-culture) in HG conditions. The levels of blood glucose in DN rats were markedly decreased and were maintained at normal levels after islet transplantation. Similarly, renal injury and microstructure damage were significantly alleviated and were accompanied by decreased apoptosis and upregulated levels of autophagy in the kidneys. However, compared with the co-cultured group (in vitro) and IT group (in vivo), highly activated expression of Notch-1 signaling weakened the therapeutic efficacy of islets co-culture or transplantation in HG conditions. Podocyte restoration was also inhibited by aberrant activation of the Notch-1 pathway in islet-transplanted rats. We also found podocyte apoptosis was slightly decreased by using a DAPT treatment, but there was no significant change in autophagy expression.

Podocyte is a kind of highly differentiated epithelial cell that attaches to the outer surface of the glomerular basement membrane and forms the final filtration barrier together to prevent protein loss^19^. Its injury and deletion is considered to be the main cause of progressive proteinuria in the patients with diabetic nephropathy^20^. Similarly, signaling abnormalities in the kidney may directly lead to podocyte injury and deletion in DN conditions. The Notch signaling pathway is an evolutionarily preserved mechanism involved in embryonic development, cellular proliferation, apoptosis and formation of many complex organ structures^21^. The protein Notch-1 has a representative receptor in the Notch protein family, and its downstream intracellular domain is deemed a key target for therapy in various kidney diseases^22^. Studies have demonstrated that aberrant activation of the Notch-1 pathway has an important role in the processes associated with podocyte injury in DN^23–25^. The NICD translocates into the nucleus and associates with DNA-binding proteins to form a DNA-bound transcription factor that can also activate expression of target genes^22,26^. Activated Notch-1 signaling can directly increase the expression level of VEGF in the kidney that is reported to be closely related to podocyte injury and progressive proteinuria. Studies have demonstrated that inhibiting VEGF and its receptors has potential therapeutic effects on podocyte injury in DN animal models^27,28^. Highly activated Notch-1 signaling and VEGF were found in HG-treated podocyte cells and DN rats. The expression of VEGF was also decreased when Notch-1 signaling was inhibited by DAPT in HG conditions. Furthermore, their expression was decreased significantly both in islets co-culture in vitro and islet transplantation in DN rats in vivo. The degree of podocyte restoration was found to be closely related to the downregulated expression of these proteins after islet transplantation in DN conditions.

Persistent and progressive proteinuria caused by losses of podocytes can further aggravate renal injury in DN patients^3,29^. Therefore, the protection and restoration of podocytes have been deemed as important for DN treatment. Owing to the limitation of conventional treatments in affecting podocyte injury, novel therapies and targeted treatments are urgently needed^30^. Many studies have demonstrated the ameliorative effects of pancreatic islet transplantation on renal injury and podocyte damage in DN animal models^31^. Renal pathological changes and microstructure damage in DN can be reversed over time by a functioning pancreatic islets graft^32^. Our previous studies also had revealed that podocyte injury could be ameliorated by islet transplantation^11^. The density of podocytes and their presence in glomeruli were significantly restored in the transplanted rats. However, in some transplanted rats therapeutic effects were not marked although blood glucose levels were maintained in the normal range. Aberrant activation of Notch-1 signaling was detected in the kidneys of these rats. Thus, we hypothesize that aberrant activation of Notch-1 signaling in the kidney could affect the therapeutic effect of islet grafts on renal injury and podocyte restoration. The results in this study also showed that highly activated Notch-1 signaling weakened improvements relating to renal injury after islet transplantation in DN conditions. Similarly, the levels of apoptosis were downregulated by inhibiting the Notch-1 pathway in HG concentrations but autophagy expression was not improved. Autophagy was markedly increased in the Jagged-1/FC + IT group, which suggests that other elements associated with a HG environment may inhibit autophagy. The imbalance between apoptosis and autophagy may affect podocyte restoration in DN conditions. The ratio of apoptosis to autophagy was proposed in this study and measured as an indicator of renal injury, and we found it was markedly decreased and accompanied with alleviated podocyte injury in DN rats after islet transplantation but still maintained at a higher level in the DAPT-treated and Jagged-1 + IT-treated DN rats. This finding may provide a new evaluation index for the degree of renal injury or recovery after treatment in the future.

There are some limitations in this study owing to the transplant site of islets. In current clinical islet transplantation, human islets are mainly transplanted into the liver through the portal vein. The islets grafts are capable of physiologic regulation based on blood glucose levels, and insulin is secreted into the liver and distributed throughout the body with blood flow^33,34^. However, with diabetes mellitus, many factors (such as aberrant signaling expressions) can disturb the homeostasis of the liver microenvironment^35–37^, which may affect the therapeutic efficacy of islet grafts *i*n vivo. The impact of liver microenvironment on transplanted islet will be evaluated in our further research, and whether the effects of islet transplantation can be improved by simulating the microenvironment in pancreas will also be studied.

**Fig. 6:**
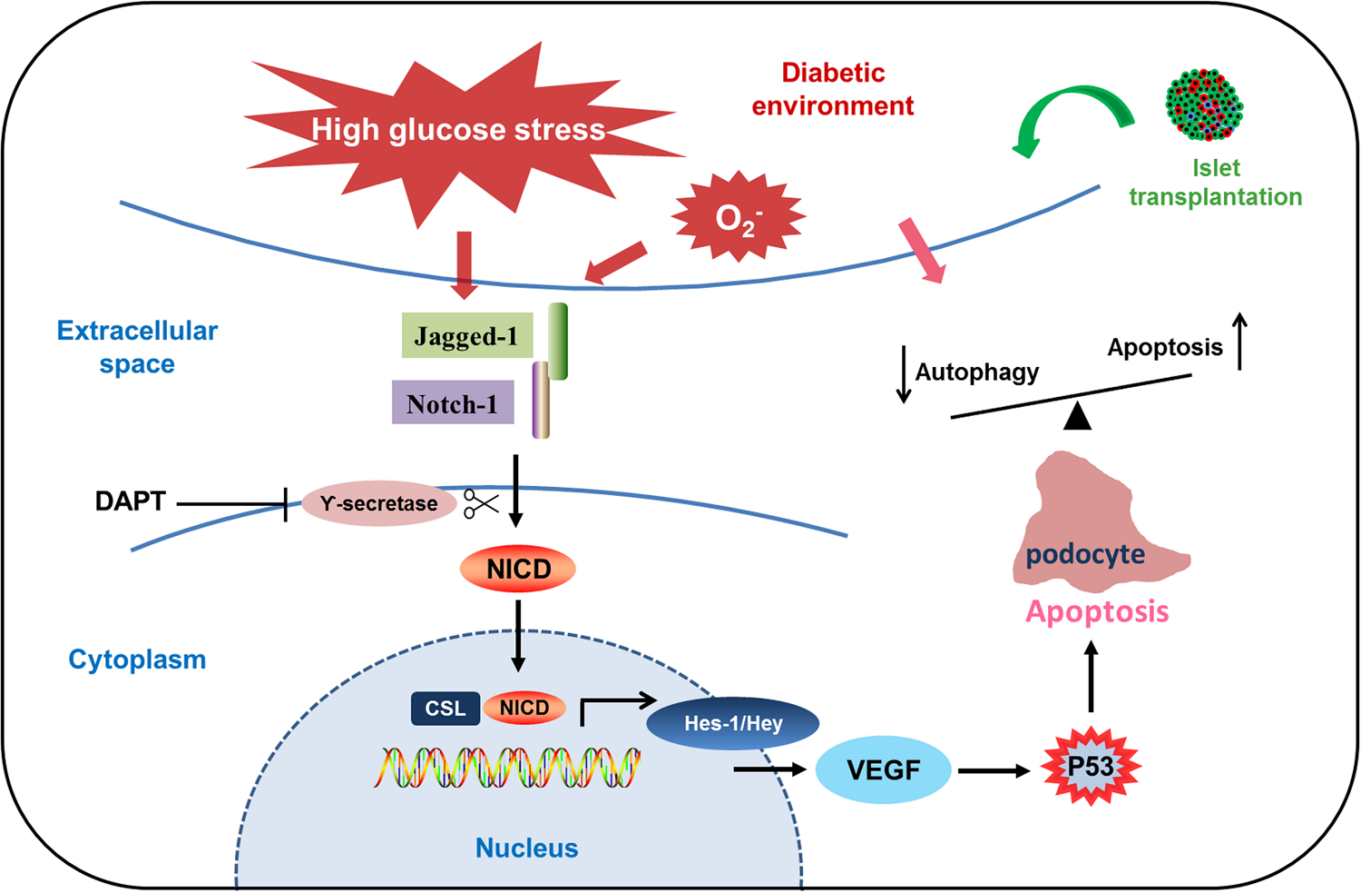
Diabetic microenvironment and aberrant activation of Notch-1 signaling affect podocyte restoration after islet transplantation by regulating the balance between autophagy and apoptosis. Islet transplantation has demonstrated its significant effects on renal damage and podocyte injury. However, in the microenvironment of diabetic nephropathy, many factors can induce podocyte injury. Notch-1 signaling can be activated by many factors, such as high-glucose stress, mechanical damages, hemodynamic changes, and oxidative stress reaction. Highly activated status of Notch-1 intracellular domain (NICD) can translocate into the nucleus and upregulate the expressions of Hes/Hey transcription factors and VEGF, which ultimately lead to podocyte apoptosis. The balance between autophagy and apoptosis of podocyte is also critical during the process of treatment of glomerular diseases and podocyte restoration. Inhibiting γ-secretase by DAPT can decrease podocyte apoptosis to some extent under the condition of DN, but it cannot upregulate the level of autophagy expression of podocytes. Therefore, the microenvironment should be taken into consideration during clinical therapies on diabetic patients through islet transplantation

In conclusion, the results in our study demonstrated significant improvement of renal injury and podocyte restoration after islet transplantation, but increased activation of the Notch-1 pathway in the kidneys weakened this therapeutic effect. This finding may suggest that the efficacy of islet transplantation could be affected by signaling abnormalities and an associated microenvironment in vivo (Fig. 6). Therefore, these factors should be taken into consideration when clinical islet transplantations are performed in different diabetic patients. This will also provide a basis for the development of individual assessments and treatments in clinical islet transplantation in different diabetic patients into the future.

## Materials and methods

### Podocytes culture and treatment

Conditionally immortalized mouse podocyte cell line (temperature-sensitive SV 40 large T-cell antigen treated) was purchased from the National Infrastructure of Cell Line Resource (Beijing, China) and cultured for proliferation at 33 °C and 5% CO_2_ in Dulbecco’s Modified Eagle Medium (DMEM, 5.6 mmol/L d-glucose, HyClone, UT, USA) supplemented with 10% fetal bovine serum (FBS; Sigma, St. Louis, MO, USA) and 10 U/mL recombinant γ-interferon (IFN-γ, Peprotech, London, United Kingdom). After passages, cells were cultured under conditions of 37 °C and 5% CO_2_ for 14 days in DMEM medium without IFN-γ to induce differentiation.

Fully differentiated podocytes were cultured in different glucose concentrations (5.6, 20.0, and 35.0 mmol/L) to investigate the degree of podocyte injury. The cells were divided into five groups after synchronization (serum-free culture for 24 h) as follows: normal glucose group (NG group, treated with 5.6 mmol/L d-glucose); high-glucose group (HG group, 35.0 mmol/L d-glucose for 48 h); Co-cultured group (podocytes were co-cultured with isolated islet cells in HG medium); DAPT group (podocytes were cultured in HG medium containing 5 μg/mL DAPT (*N*-[*N*-(3,5-Difluorophenacetyl)-l-alanyl]- S-phenylglycine t-butyl ester, Sigma Aldrich Co. CA, USA)); Co-cultured + Jagged-1 FC group (podocyte were co-cultured with islet cells in HG medium containing recombinant rat Jagged-1 FC chimera (1 μg/mL, R&D Systems, Minneapolis, MN, USA)). Also, the experiments of osmotic control to HG treatments were designed and performed in our study, including NG group (5.6 mmol/L d-glucose), Mannitol group (5.6 mmol/L d-glucose + 29.4 mmol/L d-mannitol), l-glucose group (5.6 mmol/L d-glucose + 29.4 mmol/L l-glucose) and HG group (35.0 mmol/L d-glucose).

### Animal models

A total of 50 male inbred strain Wistar rats (8 weeks, weighing 180–220 g) were provided by the Laboratory Animal Centre of Wenzhou Medical University. A total of 30 rats were used to establish DN model rats, and others were used as the negative control rats and islet donors. All rats were maintained at 24 °C ± 1 °C with a regular 12-h light/dark cycle and fed ad libitum for a week before starting the experiment. All animal experiment protocols were based on international guidelines and approved by the Animal Policy and Welfare Committee. A diabetic rat model was induced by a single intraperitoneal injection of streptozotocin (55 mg/kg, Sigma Aldrich, St. Louis, USA) in sodium citrate buffer (pH 4.5). One week later, blood glucose levels were determined by blood samples from the tail vein. Diabetic model rats were considered successfully established when the nonfasting blood glucose level was ≥ 16.67 mmol/L or more for 2 consecutive days^38^. At 12 weeks after diabetes induction, urinary microalbumin, creatinine, and urine protein were determined, and transmission electron microscopy detection was performed to assess whether rat DN models had been successfully established^39^.

Then, DN rats were randomly divided into five groups as follows: DN group (*n* = 6, established DN rats without other treatments); IT group (*n* = 6, DN rats were treated by islet transplantation under kidney capsule); DAPT group (*n* = 6, DN rats were treated by subcutaneous injection of DAPT (10 mg/kg) once a week for consecutive 8 weeks); IT + Jagged-1 FC group (*n* = 6, DN rats were treated by islet transplantation and recombinant rat Jagged-1 FC chimera administration (15 μg/mL)). The negative control group (NC group, *n* = 6) was also established.

### Islet isolation and transplantation

Islet isolation and purification were performed according to a previously described protocol^40^. The laparotomy of the donor rats was performed after anesthetization with an intraperitoneal injection of chloral hydrate. The pancreas were inflated with collagenase V and removed into a digestive tube. After digestion, the hand-picked islets were cultured in RPMI-1640 (5.6 mM glucose, Gibco, CA, USA) containing 10% (FBS, Gibco, Invitrogen, Inc, USA) at 37℃, 5% CO_2_ condition for 6 hours. Islets equivalents (IEQ) were calculated according to the diameters of the cell clusters referred to the methods reported by Lembert^41^. Then, 800–1000 IEQ islets were transplanted into the space under the kidney capsule.

### Western blot analysis

The concentration of extracted protein was detected by Protein BCA Assay (Beyotime, Jiangsu, China). Protein was separated by 10% sodium dodecyl sulfate-polyacrylamide gel electrophoresis and transferred onto nitrocellulose membranes. After blocking with nonfat milk, primary antibodies specific for Jagged-1 (Cell Signaling Technology, CST, Boston, USA), full-length Notch-1 (CST), cleaved Notch-1 (NICD, Abcam, Cambridge, UK), Synaptopodin (Abcam), Hes-1 (Abcam), VEGF (Abcam), p53 (CST), p62 (Abcam), LC3B (Abcam) were used. Goat anti-rabbit IgG conjugated with horseradish peroxidase was used to detect the expression of primary antibodies, and protein bands were visualized by ECL western blotting detection system (Amersham, Little Chalfont, UK).

### Immunohistochemistry and immunofluorescence staining

Immunohistochemical staining was performed according to the protocol described previously. Paraffin-embedded tissue sections (4 μm) were dewaxed in xylene and rehydrated in gradient alcohol. After antigen retrieval and serum blocking, the sections were incubated with primary antibody of Synaptopodin, Jagged-1, Notch-1, NICD, VEGF, P53, cleaved Caspase-3, p62, and LC3B. The positive expression was evaluated by diaminobenzidine (DAB, brown color, ZSGB-BIO, Beijing, China) after secondary antibody incubation. The immunofluorescence staining was also performed on mouse podocyte cells and renal tissues using Synaptopodin, E-cadherin, cleaved Caspase-3, and insulin polyclonal antibodies. The secondary antibodies were changed to FITC-labeled IgG (H + L) or Cy3-labeled IgG (H + L). Six regions from three separate sections were obtained from each group and all the images were analyzed on blinded slides.

### Terminal deoxynucleotidyl transferase-mediated dUTP nick end labeling (TUNEL staining)

Paraffin-embedded tissue sections (4 μm) were dewaxed in xylene and rehydrated in gradient ethanol solution. Endogenous peroxidase was blocked with hydrogen peroxide, and cell membrane was permeabilized with proteinase K. Then, the tissues were incubated with TUNEL reaction reagent in the humidified dark box. After sufficient rinsing with phosphate buffer solution (PBS) solution, the sections were treated with hematoxylin to counter stain the nucleus.

### Transmission electron microscopy

Small pieces of renal cortex tissues was obtained and fixed in 2.5% glutaraldehyde. Then, they were washed with PBS (0.01 M), and post-fixed with 1% osmium tetroxide. After gradient dehydration of acetone, the tissues were embedded in Araldite M (Sigma Aldrich). Ultrathin sections were made using an ultramicrotome (Leica, Germany), and stained with uranyl acetate and lead citrate. The sections were examined through a transmission electron microscope (H-7700, Hitachi, Japan).

### Real-time RT-PCR

Total RNA was isolated from cells or rat kidney samples with TRIzol reagent (Invitrogen, Carlsbad, CA). The relative mRNA expression levels were measured by SYBR Green qRT-PCR kit (Thermo Fisher Scientific, MA) with LightCycler real-time PCR system (Roche, Indianapolis, IN, USA). The sequences of primers used in this study were listed in Table 1.

**Table 1:** The primer sequence of each gene

Gene	Forward primer (5′ → 3′)	Reverse primer (5′ → 3′)
Mouse		
Jagged-1	CCTCGGGTCAGTTTGAGCTG	CCTTGAGGCACACTTTGAAGTA
Notch-1	GAGGCGTGGCAGACTATCATGC	CTTGTACTCCGTCAGCGTCA
NICD	CCGTGGATGACCTAGGCAAGT	TGTTGGCTCCGTTCTTCAGG
Hes-1	CCAGCCAGTGTCAACACGA	AATGCCGGGAGCTATCTTTCT
VEGF	CTGTGCAGGCTGCTGTAACG	GCTCATTCTCTCTATGTGCTGGC
P53	ATTTGTATCCCGAGTATCTG	GGTATACTCAGAGCCGGCCT
Synaptopodin	CGGAGAATCAAAACCCTCAG	CAGGACATGCCATCAGACT
β-actin	GTGGGCCGCTCTAGGCACCAA	CTCTTTGATGTCACGCACGATTTC
Rat		
Jagged-1	GGGCCAGACTGCAGGATAAAC	CGCCGTGCCCTTTGTGGAG
Notch-1	TGGCCTCAATGGATACAAATG	GGGCCAACACCACCTCAC
NICD	CCATGGTGCTGCTGTCCCGCAA	GCTTAAATGCCTCTGGAATGTG
Hes-1	GGGCAAGAATAAATGAAAG	GCGCGGTACTTCCCCAACAC
VEGF	CCTGGCTTTACTGCTGTACCT	GCTGGTAGACGTCCATGAACT
P53	GCGCACAGAGGAAGAGAA	GGCCAACTTGTTCAGTGGAG
Synaptopodin	CCAGTCACGGATGGAGAAATA	TGGCTGCTGCTTGGTGG
β-actin	TACAACTCCTTGCAGCTCC	ATCTTCATGAGGTAGTCAGTC

### Statistical analysis

All statistical analysis was performed using SPSS version 19.0 software (SPSS, Chicago, IL, USA). Statistical significance between groups was calculated with Student’s *t* test and one-way analysis of variance, and the data were presented as the mean ± standard deviation (SD). *P* values *<* 0.05 were considered significant.
